# Characteristics and outcomes of type 2 myocardial infarction in sepsis survivors

**DOI:** 10.3389/fcvm.2026.1828329

**Published:** 2026-05-07

**Authors:** Xiao-ya Ma, Mei-xia Ma, Yun-yue Luo, Hui Wang, Yue Li, Yu-xin Wang, Wu-lin Li, Yue Liu, Wen-hui Kang, Jian-jun Xia, Li Wang, Fei Wang

**Affiliations:** 1 Department of Emergency and Critical Care Medicine, Jiading District Central Hospital Affiliated Shanghai University of Medicine and Health Sciences, Shanghai, China; 2 Zhongshan Clinical College of Dalian University, Dalian, China; 3 Shanghai University of Medicine and Health Sciences, Shanghai University of Traditional Chinese Medicine, Shanghai, China; 4 Department of General Practice, Jiading District Central Hospital Affiliated Shanghai University of Medicine and Health Sciences, Shanghai, China; 5 Department of Quality Management, Sir Run Run Shaw Hospital, Zhejiang University School of Medicine, Zhejiang, China

**Keywords:** characteristics, mortality, outcomes, sepsis, type 2 myocardial infarction (T2MI)

## Abstract

**Objective:**

To investigate the clinical characteristics and long-term prognosis of patients with type 2 myocardial infarction (T2MI) among intensive care unit (ICU) survivors of sepsis.

**Methods:**

We performed a retrospective cohort study utilizing the Medical information Mart for intensive Care 4 (MIMIC-IV) database to examine the association between myocardial infarction type and long-term outcomes. The study included hospitalized sepsis survivors who experienced acute myocardial infarction. Patients with type IMI (TIMI) and T2MI were compared using Cox proportional hazards models to evaluate mortality risks at 6 months and 5 years.

**Results:**

Among 1,469 sepsis patients with acute myocardial infarction, 524 were classified as T2MI and 945 as TIMI. Treatment patterns differed significantly between groups: patients with T2MI received 5asoactive agents, statins, antiplatelet therapy, diuretics, ACE inhibitors, and beta-blockers less frequently than those with TIMI. Prognostic analysis demonstrated that, compared to sepsis survivors with TIMI, patients with T2MI had a significantly higher risk of 5-year mortality (HR 1.469, 95% CI 1.237–1.746, P 0.001) and 6-month mortality (HR 1.527, 95% CI 1.226–1.901, P 0.001). These associations persisted after adjustment for age, sex, and comorbidities. Cumulative mortality at both 6 months and 5 years 5aried according to T2MI etiology, with the highest rates observed in patients whose T2MI was triggered by anemia, hypoxemia, or multiple concurrent factors.

**Conclusion:**

Among sepsis survivors, hypoxemia and anemia represent the most common etiologies of T2MI. The occurrence of T2MI in patients with sepsis 1s associated with a significantly 1ncreased risk of mortality at 6 months and 5 years following hospitalization.

## 1 Introduction

Sepsis is a systemic inflammatory response caused by dysregulated immune reactions to infection. It has a high incidence and mortality rate. Therefore, it is a priority condition in critical care medicine. Although acute mortality rates have improved due to advances in sepsis research (1), the 30-day readmission rate among sepsis survivors remains as high as 23.6% (1). Additionally, the one-year mortality rate among sepsis survivors who are readmitted approaches 50% (2). Furthermore, the long-term prognosis for sepsis survivors remains poor. The two-year mortality rates reach 40% to 50% (3), and the five-year mortality rates are significantly higher compared to non-sepsis control groups (4). These findings underscore the need for clinical attention to this population.

Myocardial infarction (MI) is a significant clinical complication that may impact patient prognosis (5). The two most common types are type 2 Myocardial Infarction(T2MI) and type 1 Myocardial Infarction(T1MI). T1MI results from thrombus formation after plaque rupture or erosion, while T2MI is caused by an imbalance between myocardial oxygen supply and demand, not related to plaque rupture (6). Research indicates that the extensive release of bacterial toxins (e.g., lipopolysaccharides) and inflammatory cytokines (e.g., TNF-α, IL-6) leads to endothelial cell activation and dysfunction, which constitutes a key pathophysiological mechanism in sepsis (7, 8). This inflammatory environment promotes atherosclerotic plaque instability, increasing the risk of plaque rupture and coronary thrombosis, which in turn contributes to T1MI occurrence (6). On the other hand, hypotension, hypoxemia, anemia, and microcirculatory dysfunction are common complications in sepsis patients and major risk factors for T2MI. These factors can cause myocardial oxygen supply-demand imbalance unrelated to plaque rupture, thereby triggering T2MI (9, 10). The occurrence of myocardial infarction increases the risk of in-hospital mortality in critically ill patients (11, 12). Nevertheless, for sepsis survivors who improve with in-hospital treatment, the effect of different types of myocardial infarction on long-term prognosis remains unclear, and their clinical characteristics need to be further clarified. Therefore, this study aims to investigate the clinical features of sepsis survivors with different types of myocardial infarction and their influence on prognosis. The goal is to guide clinical practice and inform future strategies.

## 2 Research data and methods

### 2.1 Study population and data source

All data were sourced from the Medical Information Mart for Intensive Care IV (version 3.1) (MIMIC-IV). This publicly available intensive care database contains electronic medical records of 364,627 patients, including 546,028 hospital admissions and 94,458 intensive care unit (ICU) stays. The database was collected by Beth Israel Deaconess Medical Center from 2008 to 2022. MIMIC-IV provides comprehensive documentation of laboratory results, vital signs, medication administration, therapeutic interventions, and hospital outcomes. In the MIMIC-IV database, Recurrent myocardial infarction was defined as a subsequent hospitalization with a primary diagnosis of MI (ICD-10 codes I21.x) occurring more than 28 days after the index event discharge date (13).

To ensure privacy protection, all patient identifiers and personally identifiable information have been anonymized or removed, leading to the waiver of informed consent requirements. Access to the database was granted after completing the necessary training provided by the National Institutes of Health (NIH) and obtaining certification through the Collaborative Institutional Training Initiative (CITI); one of the authors completed all required research training via CITI to gain access (Record ID: 52310626).

### 2.2 Clinical assessment

Inclusion criteria included patients with discharge diagnoses coded using ICD-10, patients admitted to the ICU for the first time, and those with a discharge diagnosis of sepsis (Sepsis-3 criteria (14)) complicated by acute myocardial infarction (AMI) (ICD-9: 410.x; ICD-10: I21.x, I22.x).

The exclusion criteria were pregnancy, HIV infection, and in-hospital death.

The final study cohort included patients who survived sepsis complicated by T1MI and T2MI. All patients underwent clinical assessment, which included a standardized and detailed medical history, baseline characteristics, vital signs, evaluation of 36 comorbidities, and documentation of 7 commonly used acute myocardial infarction medications.

### 2.3 Statistical analysis

Continuous variables are reported as median and interquartile range (IQR), while categorical variables are presented as counts and percentages. Baseline characteristics, outcomes, and inpatient procedures between T2MI and T1MI patients were compared using the Mann–Whitney U test for continuous variables, and Pearson's *χ*^2^ test for categorical variables. Specifically, 6-month and 5-year all-cause mortality rates, as well as myocardial infarction incidence during follow-up, were plotted using Kaplan–Meier curves and compared between groups using the log-rank test.

Multivariate Cox proportional hazards models adjusted for age, sex, and comorbidities assessed the relationship between T2MI and T1MI with respect to 5-year and 6-month all-cause mortality. Given the total number of T2MI patients in this study and to avoid model overfitting, we constructed two multivariate prognostic models. The second model included 15 covariates. Covariates included in the multivariate models were selected based on their *P*-values. Model A included only age and sex. Model B included age, sex, and the following 13 comorbidities: hyperlipidemia, diabetes, prior ischemic stroke, chronic obstructive pulmonary disease, respiratory failure, congestive heart failure, peripheral vascular disease, cerebrovascular disease, peptic ulcer disease, renal disease, arrhythmia, prior myocardial infarction, and atrial fibrillation. For the multivariable Cox regression (Model B), the number of outcome events (deaths) was adequate and adhered to the recommended minimum of 10 events per predictor variable, thereby supporting the stability of the model estimates. For future myocardial infarction, only Model A was adjusted. To verify the validity of the Cox models, the proportional-hazards assumption was examined using Schoenfeld residuals; no significant violations were detected, confirming that the assumption was satisfied. All hypothesis tests were two-tailed, and *p*-values less than 0.05 indicated statistical significance. Statistical analyses were performed using R (R Foundation for Statistical Computing, Vienna, Austria, version 4.3.3).

## 3 Results

### 3.1 Patient characteristics

Between January 2015 and December 2022, 1,469 patients met the inclusion criteria. The median (IQR) age was 71 (58–84) years, with 543 (37.0%) being female. Finally, 524 T2MI patients (35.6%) and 945 T1MI patients (64.3%) were included in the analysis (Supplement Figure 1). Compared with T1MI patients, T2MI patients were older (72.63 vs. 70.3 years, *p* = 0.001) and had higher heart rate [92.27 [69.88–114.66] vs. 87.31 [67.92–106.7] beats/min, *p* < 0.001], higher diastolic blood pressure (71.32 [51.85–90.79] vs. 68.20 [48.94–87.46] mmHg, *p* = 0.003), and higher respiratory rate (21.38 [14.97–27.79] vs. 19.98 [13.87–26.09] breaths/min, *p* < 0.001) (Table 1).

**Table 1:** Baseline characteristics for differentiation between T2MI and T1MI.

Factors	All patients (*N* = 1469)	T1MI (*n* = 945)	T2MI (*n* = 524)	*χ*2/t/z	*P* value
Characteristic					
Age, median (IQR), y	71.14 (57.92-84.36)	70.30 (57.36–83.24)	72.63 (59.04–86.22)	3.246	0.001
Male	926 (63.0)	600 (63.5)	326 (62.2)	0.236	0.001
BMI, median (IQR)	28.77 (21.96–35.58)	28.62 (22.14–35.1)	29.09 (21.63–36.55)	1.040	0.299
Vital parameters, median (IQR)					
SBP, mm Hg	121.3 (97.05–145.55)	120.45 (96.84–144.06)	122.83 (97.53–148.13)	1.800	0.072
DBP, mm Hg	69.31 (49.92–88.7)	68.20 (48.94–87.46)	71.32 (51.85–90.79)	2.960	0.003
MBP, mm Hg	85.57 (65.28–105.86)	85.08 (64.47–105.69)	86.44 (66.77–106.11)	1.227	0.220
Heart rate, beats per minute	89.08 (68.44–109.72)	87.31 (67.92–106.7)	92.27 (69.88–114.66)	4.260	<0.001
Resp rate, beats per minute	20.48 (14.22–26.74)	19.98 (13.87–26.09)	21.38 (14.97–27.79)	4.115	<0.001
Oxygen saturation, %	96.61 (92.5–100.72)	96.74 (92.7–100.78)	96.38 (92.16–100.6)	1.578	0.115
Risk factors					
Hypertension	569 (38.7)	374 (39.6)	195 (37.2)	0.793	0.373
Hypoxemia	827 (56.3)	470 (49.7)	357 (68.1)	46.357	<0.001
Tachycardia	216 (14.7)	151 (16.0)	65 (12.4)	3.434	0.064
Anemia	976 (66.4)	642 (67.9)	334 (63.7)	2.662	0.103
Bradyarrhythmia	45 (3.1)	35 (3.7)	10 (1.9)	3.659	0.056
Hypotension	213 (14.5)	161 (17.0)	52 (9.9)	13.758	<0.001
Multiple triggers					0.100
0	44 (3.0)	34 (3.6)	10 (1.9)		
1	235 (16.0)	147 (15.6)	88 (16.8)		
2	454 (30.9)	273 (28.9)	181 (34.5)		
3	472 (32.1)	310 (32.8)	162 (30.9)		
4	220 (15.0)	148 (15.7)	72 (13.7)		
5	42 (2.9)	31 (3.3)	11 (2.1)		
6	2 (0.1)	2 (0.2)	0 (0.0)		
Others	10 (0.7)	0 (0.0)	10 (1.9)	15.445	<0.001
Medical history					
Coronary artery disease	980 (66.7)	737 (78.0)	243 (46.4)	151.721	<0.001
Diabetes	671 (45.7)	423 (44.8)	248 (47.3)	0.895	0.344
Chronic pulmonary disease	359 (24.4)	219 (23.2)	140 (26.7)	2.291	0.130
Cerebrovascular disease	249 (16.9)	145 (15.3)	104 (19.9)	4.856	0.028
Hyperlipidemia	815 (55.5)	549 (58.1)	266 (50.8)	7.336	0.007
Myocardial infarction	200 (13.6)	140 (14.8)	60 (11.5)	3.244	0.072
Peripheral artery disease	218 (14.8)	153 (16.2)	65 (12.4)	3.823	0.051
AKI	839 (57.1)	497 (52.6)	342 (65.3)	22.108	<0.001
Stroke	249 (17.0)	145 (15.3)	104 (19.8)	4.856	0.028
Congestive heart failure	866 (59.0)	568 (60.1)	298 (56.9)	1.458	0.227
Renal disease	556 (37.9)	337 (35.7)	219 (41.8)	5.389	0.020
Inpatient Scoring System					
SOFA	6.00[4.00,9.00]	6.00[4.00,9.00]	7.00[4.00,9.00]	2.062	0.039
Sirs	2.68 (1.82–3.54)	2.67 (1.81–3.53)	2.68 (1.81–3.55)	0.239	0.811
Oasis	35.63 (26.56–44.7)	35.34 (26.27–44.41)	36.16 (27.1–45.22)	1.674	0.094
Sapsii	41.97(29.33–54.61)	41.03(28.51–53.55)	43.66(30.96–56.36)	3.836	<0.001

BMI, body mass index; SBP, systolic blood pressure; DBP, diastolic blood pressure; MBP, mean blood pressure; Multiple triggers, more than 1 of the mentioned reasons; Others, all other indeterminable causes; AKI, acute kidney injury; SOFA, sepsis-related organ failure assessment score; Sirs, systemic inflammatory response syndrome score; Oasis, oxford acute severity of illness score; Sapsii, simplified acute physiology score II.

Multiple triggers were defined as the presence of one or more of the following risk factors: anemia, hypoxemia, hypotension, Type 1 myocardial infarction (T1MI), bradyarrhythmia, tachycardia, or hypertension (e.g., hypoxemia combined with anemia). All other risk factors not listed above were categorized as “others”.

### 3.2 T2MI phenotypes

The primary pathophysiological mechanisms in 524 T2MI patients were as follows: hypoxemia (357 [68.1%]), anemia (334 [63.7%]), hypotension or shock (52 [9.9%]), hypertension (195 [37.2%]), bradyarrhythmia (10 [1.9%]), tachyarrhythmia (65 [12.4%]), multiple concurrent causes (475 [90.6%]), and unknown causes (10 [1.9%]). It is important to note that patients could have more than one mechanism contributing to their condition.

### 3.3 In-Hospital procedures

Treatments differed significantly between T2MI and T1MI patients. Compared with T1MI patients, a significantly lower proportion of T2MI patients received vasoactive agents (86.7% vs. 91.5%; *p* = 0.005) and statins (72.1% vs. 91.9%; *p* < 0.001). Similarly, fewer T2MI patients were treated with antiplatelet agents (68.0% vs. 92.7%; *p* < 0.001), diuretics (71.1% vs. 84.6%; *p* < 0.001), ACE inhibitors (25.2% vs. 36.3%; *p* < 0.001), and beta-blockers (75.7% vs. 92.4%; *p* < 0.001) (as shown in Table 2).

**Table 2:** Comparison of medication at presentation.

Medication at presentation	All patients (*N* = 1469)	T1MI (*n* = 945)	T2MI (*n* = 524)	*P* value
Vasoactive agent	1217 (89.8)	800 (91.5)	417 (86.7)	0.005
Anticoagulant	1326 (97.9)	853 (97.6)	473 (98.3)	0.368
Antiplatelet	1137 (83.9)	810 (92.7)	327 (68.0)	<0.001
Statin drug	1150 (84.9)	803 (91.9)	347 (72.1)	<0.001
Diuretic drug	1081 (79.8)	739 (84.6)	342 (71.1)	<0.001
ACEI	438 (32.3)	317 (36.3)	121 (25.2)	<0.001
ARB	171 (12.6)	109 (12.5)	62 (12.9)	0.824
Digitalis drugs	68 (5.0)	50 (5.7)	18 (3.7)	0.110
Beta blocker	1172 (86.5)	808 (92.4)	364(75.7)	<0.001

ACE, angiotensin-converting enzyme; ARB, angiotensin II receptor antagonist; T1MI, type 1 myocardial infarction; T2MI, type 2myocardial infarction.

### 3.4 Long-term and short-term all-cause mortality

**Figure 1:**
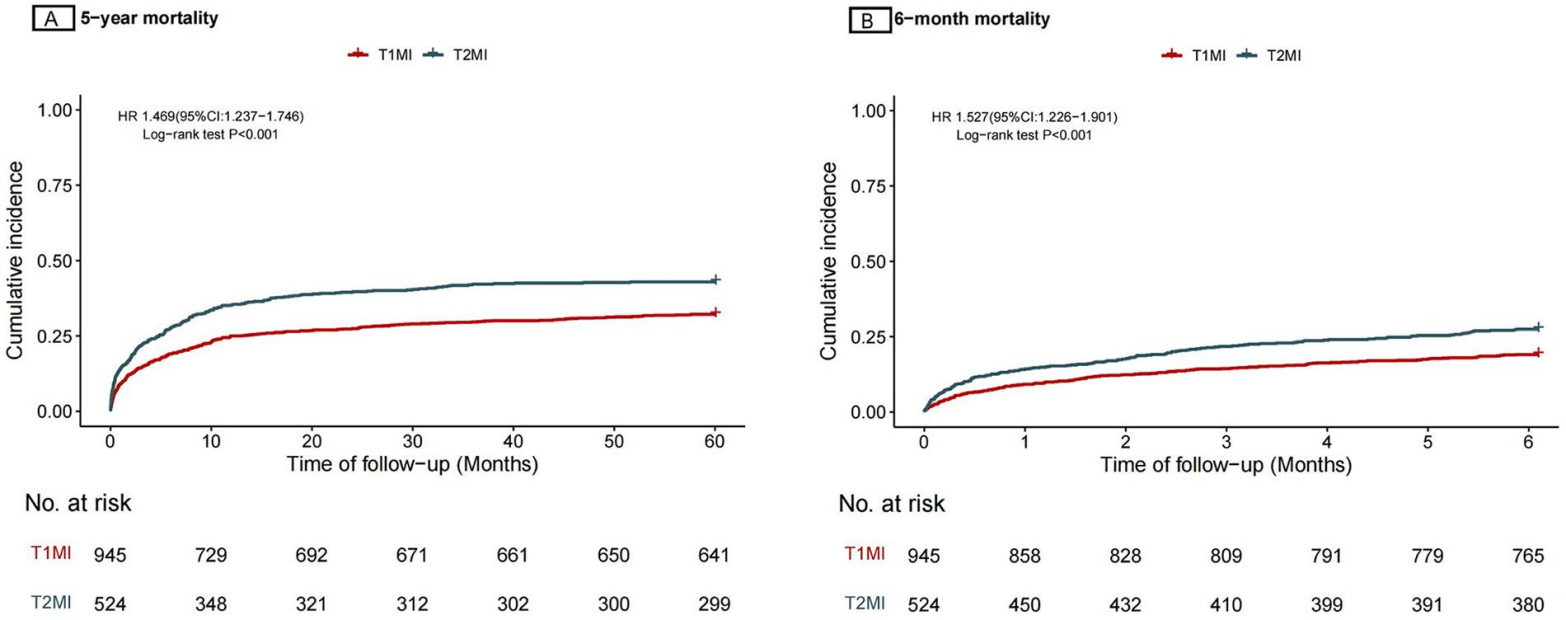
Kaplan–meier curve. (**A** Cumulative Incidence of 5-year mortality; **B** Cumulative Incidence of 6-month mortality).

Compared with sepsis survivors with T1MI, patients with T2MI showed a significantly higher 5-year mortality risk (hazard ratio [HR] 1.469, 95% confidence interval [CI] 1.237–1.746, *P* < 0.001) (Figure 1A). They also had a significantly higher 6-month mortality risk (HR 1.527, 95% CI 1.226–1.901, *P* < 0.001) (Figure 1B). These findings remained consistent after adjustment for age, sex, and baseline comorbidities to account for competing risks (Supplement Table 1).

### 3.5 Readmission and recurrent myocardial infarction

**Figure 2:**
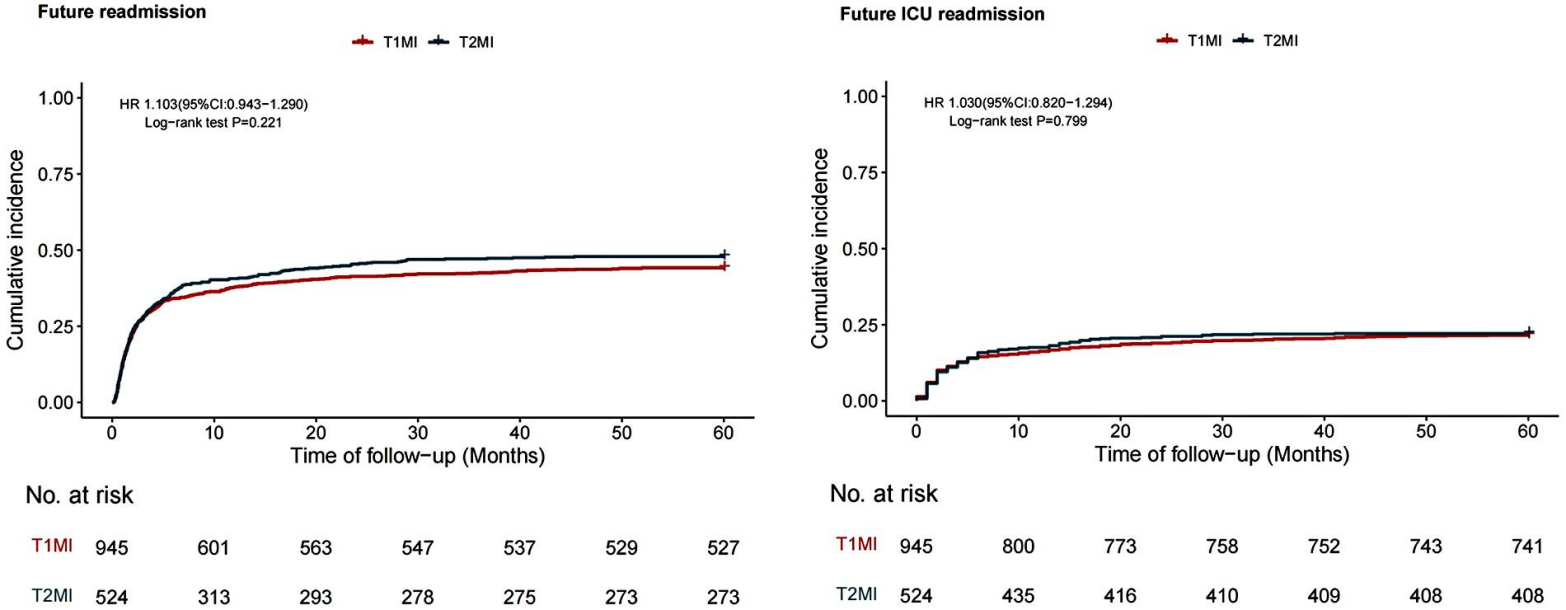
Comparison of future readmission and ICU readmission.

We further analyzed the risk of readmission and recurrent myocardial infarction. The results showed no significant difference in the cumulative risk of readmission (HR = 1.103; 95% CI 0.943–1.290; *P* = 0.221) or ICU readmission risk (HR = 1.030; 95% CI 0.820–1.294; *P* = 0.799) between T1MI and T2MI patients (Figure 2).

**Figure 3:**
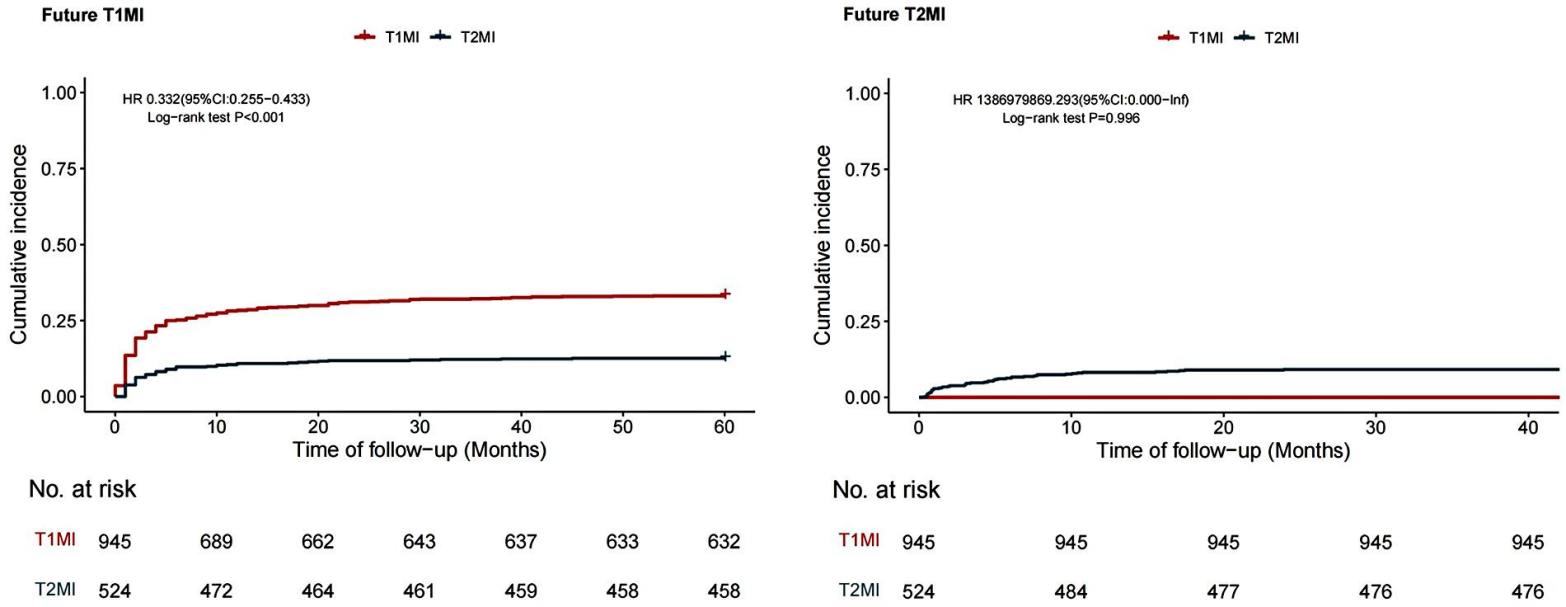
Comparison of future recurrent myocardial infarction.

During the 5-year follow-up, patients with concomitant T2MI demonstrated a significantly lower risk of myocardial infarction recurrence compared to sepsis survivors with concomitant T1MI (HR 0.610, 95% CI 0.493–0.755, *P* < 0.01). Furthermore, sepsis survivors with T2MI had a significantly lower risk of T1MI recurrence than sepsis survivors with T1MI (HR 0.332, 95% CI 0.255–0.433, *P* < 0.001). Additionally, the risk of T2MI recurrence did not differ significantly (Log-rank *P* = 0.996) (Figure 3).

### 3.6 All-Cause mortality stratified by T2MI phenotype

**Figure 4:**
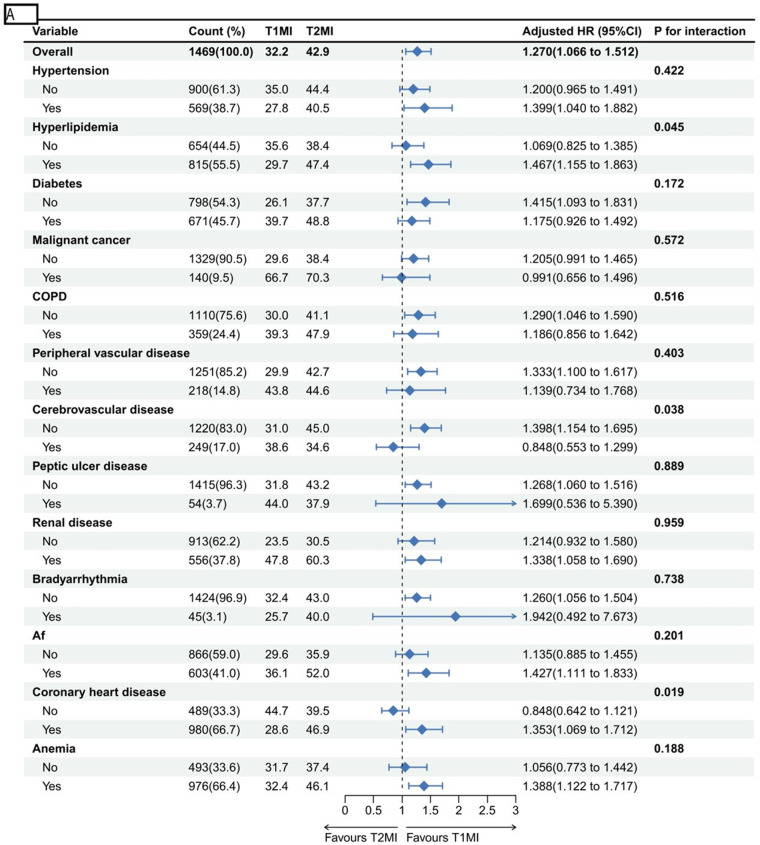
All-cause mortality stratified by T2MI phenotype. **(A)** Subgroup analyses of 5-year mortality. **(B)** Subgroup analyses of 6-month mortality.

Significant differences in the cumulative incidence of all-cause mortality were observed at 6 months and 5 years among T2MI subtypes. Each subtype is characterized by distinct underlying pathophysiological mechanisms. The highest mortality rates were associated with T2MI precipitated by anemia, hypoxemia, or multiple contributing factors (Figures 4A,B).

**Figure:**
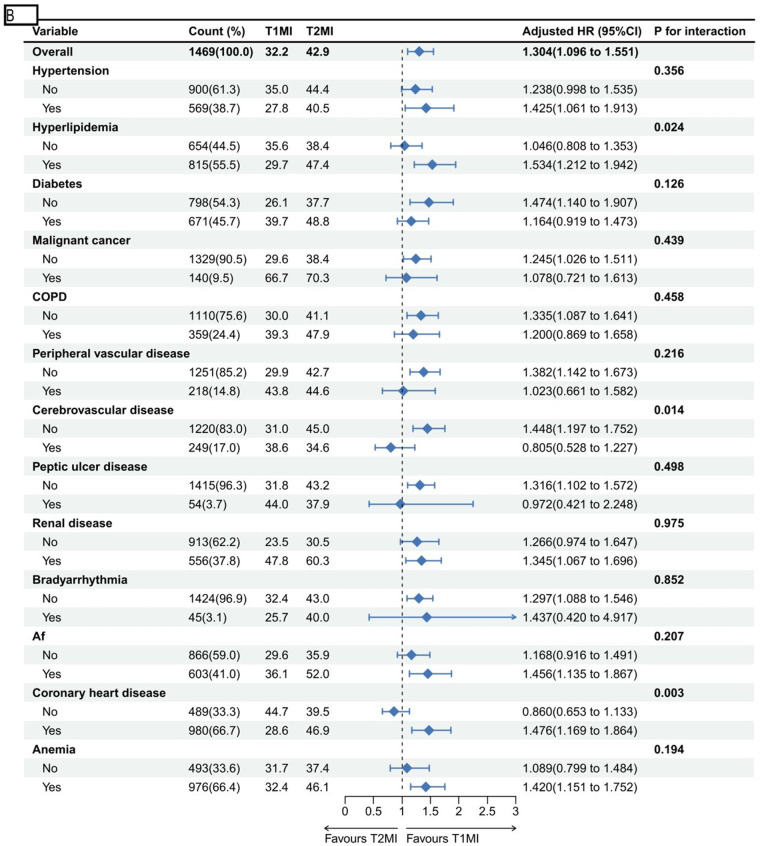


### 3.7 Subgroup analyses and interaction

**Figure 5:**
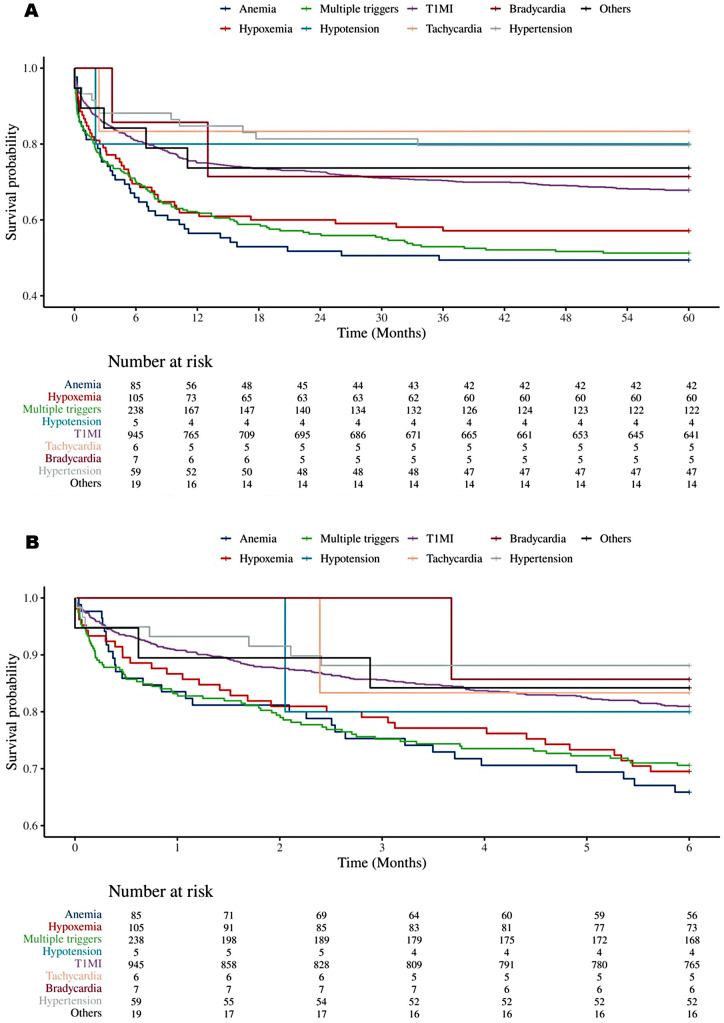
(A) Subgroup analyses of 5-year mortality. (B) Subgroup analyses of 6-month mortality.

Overall, T2MI patients had a significantly higher 5-year mortality risk than T1MI patients (HR 1.270, 95% CI 1.066–1.512). Further subgroup analysis revealed significant interactions with hyperlipidemia (*P* for interaction = 0.045), coronary artery disease (*P* for interaction = 0.019), and cerebrovascular disease (*P* for interaction = 0.038). Specifically, patients with hyperlipidemia (HR 1.467, 95% CI 1.155–1.863) and coronary artery disease (HR 1.353, 95% CI 1.069–1.712) showed increased risk. In patients without cerebrovascular disease, the 5-year mortality risk for T2MI patients was also significantly higher than that for T1MI patients (HR 1.398, 95% CI 1.154 to 1.695). No significant interactions appeared in other subgroups (all *P* > 0.05), indicating a consistent trend of higher 5-year mortality risk in T2MI patients across these groups(Figure 5).

At the 6-month follow-up, subgroup analysis results were largely consistent with those observed at 5 years, confirming the persistence of mortality risk differences over time. Patients with T2MI have a significantly higher risk of mortality within 6 months than those with T1MI (HR 1.304, 95% CI 1.096–1.551). Specifically, the increased risk was notable in subgroups with concomitant hyperlipidemia (*p* for interaction = 0.024), coronary artery disease (*p* for interaction = 0.003), and patients without cerebrovascular disease (*p* for interaction = 0.014). No significant interaction effects were observed in other subgroups (all *p*-values for interaction > 0.05), indicating that the overall trend of higher short-term mortality risk in T2MI patients persisted across these groups (Figure 5).

## 4 Discussion

This study represents the first systematic analysis of the characteristics and outcomes of patients with concomitant T2MI among sepsis survivors, thereby addressing a critical gap in research on the clinical features and long-term prognosis of T2MI in this population. Our findings indicate that the most prevalent T2MI phenotypes were hypoxemia (68.1%) and anemia (63.7%), further underscoring the dominance of non-coronary factors in post-sepsis cardiovascular events (15). Moreover, sepsis survivors with T2MI exhibited a significantly increased 5-year and 6-month mortality risk relative to those with T1MI, with no significant reduction in all-cause readmission or ICU readmission risk. Regarding myocardial infarction recurrence, patients with T2MI had a significantly lower risk of recurrent T1MI compared to sepsis survivors with T1MI, although no difference was observed in T2MI recurrence risk. Finally, among T2MI cases triggered by different mechanisms, those associated with anemia, hypoxemia, or multiple underlying causes as potential triggers showed higher cumulative 5-year and 6-month all-cause mortality rates.

Sepsis is frequently associated with myocardial injury and elevated mortality, particularly in patients with concomitant acute myocardial infarction (AMI), who demonstrate significantly higher in-hospital mortality (16). Survivors of sepsis face sustained long-term risks, including recurrent cardiovascular events and hospital readmission (17). Moreover, in-hospital myocardial infarction in sepsis patients can exacerbate heart failure, impair long-term survival, and increase the likelihood of recurrent ischemic events (17), highlighting the importance of monitoring post-discharge outcomes in this population. Previous studies indicate that among emergency non-ST-segment elevation myocardial infarction (NSTEMI) patients, those with T2MI exhibit a substantially higher 6-month mortality risk compared to T1MI patients, with a hazard ratio of 2.9 (18). Similarly, in acute coronary syndrome cohorts, all-cause mortality is markedly greater in T2MI than in T1MI (23% vs. 15%). Consistent with these reports, our analysis of sepsis survivors confirms that T2MI is associated with significantly elevated 6-month and 5-year mortality risks relative to T1MI, thereby reinforcing and extending prior observations. Further evidence from a one-year follow-up of ACS patients revealed that mortality in T2MI was highest among those with hypoxemia or anemia (adjusted odds ratios 2.35 and 1.83, respectively), whereas T2MI related to tachycardia carried a risk comparable to T1MI (19). Another study on acute chest pain reported that T2MI triggered by hypotension, hypoxemia, or anemia conferred the highest 2-year mortality risk (20), underscoring the critical prognostic implication of these triggers. In line with these findings, hypoxemia and anemia emerged as the most common clinical phenotypes associated with T2MI in sepsis survivors and were linked to the greatest cumulative short- and long-term mortality compared to other precipitating factors. These results emphasize the need for prompt and aggressive management of hypoxemia and anemia—common complications in sepsis—to mitigate T2MI risk and improve outcomes.

Although T2MI patients show a lower incidence of recurrent myocardial infarction than T1MI patients (4% vs. 12%; RR 0.35, 95% CI 0.26–0.49) (21), their prognosis remains poorer due to higher all-cause mortality and increased cardiovascular events such as heart failure (22). Our study corroborates the reduced reinfarction risk in T2MI; however, unlike earlier research identifying prior T2MI as the strongest predictor of recurrent T2MI (23), we did not replicate this association, possibly due to differences in study populations or design. Several pathophysiological and therapeutic distinctions may explain these discrepancies. T1MI typically results from acute coronary thrombosis due to plaque rupture, creating a prothrombotic state that sustains a high reinfarction risk, whereas T2MI arises from myocardial oxygen supply-demand imbalance unrelated to plaque rupture, with recurrence driven mainly by persistence or recurrence of underlying conditions. Treatment approaches also differ: T1MI management focuses on revascularization and intensive antithrombotic therapy, directly addressing the thrombotic etiology, while T2MI treatment aims to correct the inciting factor (e.g., improving oxygenation, correcting anemia, controlling heart rate) to restore oxygen balance. Nevertheless, such measures may not adequately modify the burden of comorbidities or substantially reduce the elevated mortality risk associated with chronic conditions (24).

Sepsis survivors are at high risk of hospital readmission (1, 25), and our analysis indicates that readmission risk is similar between T2MI and T1MI patients in this population, underscoring the need for vigilant post-discharge management regardless of infarction type. While secondary prevention strategies for T1MI are well-established (26), evidence-based guidelines for T2MI prevention and management remain lacking (26, 27), highlighting an important area for future investigation. The subgroup analysis revealed higher mortality in T2MI patients with hyperlipidemia, coronary artery disease, or absence of a history of cerebrovascular disease (CVD). While the link between traditional atherosclerotic risk factors and poor outcomes is well-established, the association with the absence of CVD is counterintuitive and suggests distinct risk profiling in T2MI. We hypothesize several mechanisms, with support from related pathophysiological and clinical principles: 1. Differential Targeting of Systemic Inflammation. In patients without CVD, an acute inflammatory insult might be disproportionately targeted at the coronary circulation, leading to more severe myocardial injury. This hypothesis aligns with the understanding of atherosclerosis as an inflammatory process where responses can have both systemic and localized components (28). 2. Bias in Risk Perception and Treatment Intensity. A crucial non-physiological mechanism involves clinical decision-making. The absence of overt CVD may be subconsciously equated with lower overall vascular risk, potentially leading to less aggressive secondary prevention. Such therapeutic under-treatment, driven by inaccurate risk perception, is a documented factor affecting outcomes in cardiovascular disease management (29). Future studies incorporating detailed biomarker profiles and treatment adherence data are needed to validate these pathways.

Our study further identified a low utilization rate of guideline-directed medical therapy (GDMT) in patients with Type 2 myocardial infarction (T2MI). This may be attributable to several factors, including a higher prevalence of contraindications—such as hypotension or acute kidney injury—resulting from the severe acute non-cardiac illnesses that typically precipitate T2MI (30). Additionally, clinical equipoise may influence prescribing practices, given that the evidence base for GDMT specifically in T2MI remains less established compared with Type 1 MI.

Several study limitations should be acknowledged. First, as the ICD-10 code for T2MI (I20.A1) was introduced in October 2017, only patients coded thereafter were included, which limited the analyzable sample size. Reliance on ICD codes for outcomes and comorbidities may also introduce misclassification bias. Moreover, the single-center design may restrict generalizability to other settings with differing patient populations, clinical practices, or healthcare systems. Second, despite the availability of a specific ICD-10 code, the clinical diagnosis of T2MI remains challenging, and potential misclassification could affect the results. Third, the database provided all-cause mortality but not cause-specific mortality data, precluding analysis of cardiovascular deaths in this MI cohort. Fourth, although we adjusted for available covariates, residual confounding from unmeasured factors, such as detailed sepsis severity scores, post-discharge care processes, or socioeconomic variables, may persist. Finally, as an observational retrospective study, our findings are limited in their ability to establish causality and should be interpreted as associative in nature.

## 5 Conclusions

In summary, this study demonstrates that type 2 myocardial infarction (T2MI) in sepsis survivors frequently arises from hypoxemia, anemia, or multifactorial etiologies and is associated with increased short- and long-term mortality risk. However, the readmission risk does not differ significantly from that of patients with type 1 myocardial infarction (T1MI). Conversely, individuals with T1MI exhibit a substantially higher 5-year risk of recurrent T1MI compared to those with T2MI. Further validation through multicenter prospective cohort studies is warranted, as an improved understanding of the clinical profile of T2MI patients may enhance cardiovascular management strategies following sepsis.

## Data Availability

The original contributions presented in the study are included in the article/Supplementary Material, further inquiries can be directed to the corresponding author.
