# Supplementary material for: Characteristics and outcomes of type 2 myocardial infarction in sepsis survivors
Source: Front Cardiovasc Med. 2026 May 7;13:1828329. doi: 10.3389/fcvm.2026.1828329 (PMC13190167; doi:10.3389/fcvm.2026.1828329)
Supplement: Supplementary file 1 [file Datasheet1.docx]

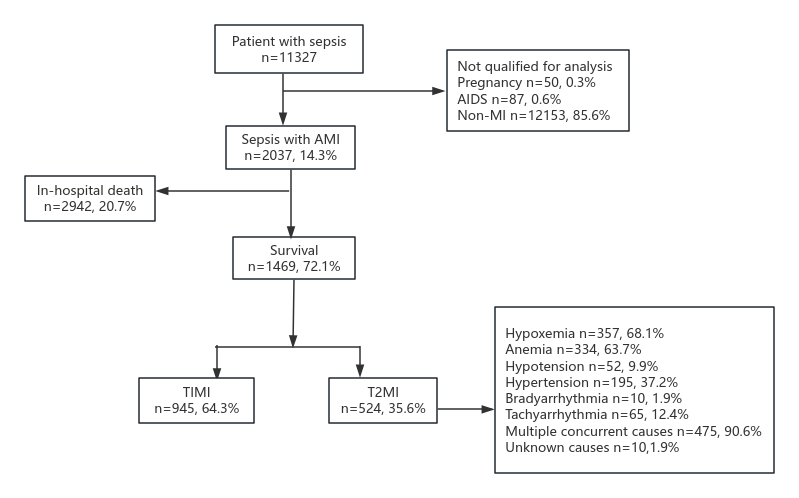


**Supplement Figure 1.** Patient flow diagram

**Supplement Table 1.** Multivariable Cox proportional hazards models with adjustments

| 5-year all-cause mortality | | | |
| --- | --- | --- | --- |
|  | **Crude Model** | **Model A** | **Model B** |
| T2MI, HR(95%CI) | 1.469(1.237~1.746) | 1.333(1.121~1.586) | 1.256(1.050~1.501) |

| 6-month all-cause mortality | | | |
| --- | --- | --- | --- |
|  | **Crude Model** | **Model A** | **Model B** |
| T2MI, HR(95%CI) | 1.527(1.226~1.901) | 1.352(1.084~1.686) | 1.274(1.015~1.598) |

HR=Hazard Ratio, 95% CI= 95% Confidence Interval,

Model A= adjusted for age and sex,

Model B= adjusted for age, sex, hyperlipidemia, diabetes, cerebral infarction, COPD, respiratory failure, congestive heart failure, peripheral vascular disease, cerebrovascular disease, peptic ulcer disease, renal disease, arrhythmia,old MI, Atrial fibrillation.
